# Updated molecular epidemiology of carbapenem-non-susceptible *Escherichia coli* in Taiwan: first identification of KPC-2 or NDM-1-producing *E. coli* in Taiwan

**DOI:** 10.1186/1471-2334-13-599

**Published:** 2013-12-20

**Authors:** Ling Ma, L Kristopher Siu, Jung-Chung Lin, Tsu-Lan Wu, Chang-Phone Fung, Jann-Tay Wang, Po-Liang Lu, Yin-Ching Chuang

**Affiliations:** 1 National Institutes of Infectious Diseases and Vaccinology, National Health Research Institutes, Miaoli, Taiwan; 2 Division of Infectious Diseases and Tropical Medicine, Department of Internal Medicine, Tri-Service General Hospital, National Defense Medical Center, Taipei, Taiwan; 3 Department of Clinical Pathology, Linkou Chang Gung Memorial Hospital, Taoyuan, Taiwan; 4 Section of Infectious Diseases, Department of Medicine, Taipei Veterans General Hospital, Taipei, Taiwan; 5 Division of Infectious Diseases, Department of Medicine, National Taiwan University Hospital, Taipei, Taiwan; 6 Department of Internal Medicine, Kaohsiung Medical University Hospital, 100 Tzyou 1st Road, Kaohsiung City, Taiwan; 7 College of Medicine, Kaohsiung Medical University, 100 Tzyou 1st Road, Kaohsiung City, Taiwan; 8 Department of Internal Medicine and Medical Research, Chi Mei Medical Center, Tainan, Taiwan; 9 Department of Internal Medicine, Chi Mei Medical Center, Liouying, Tainan, Taiwan

## Abstract

**Background:**

The global spread and increasing incidence of carbapenem-resistant Enterobacteriaceae have resulted in treatment and public health concerns. Here, we present an investigation of the molecular mechanisms and clonality of carbapenem-non-susceptible *Escherichia coli* (CnSEC) based on a nationwide survey in Taiwan.

**Methods:**

We collected 32 and 43 carbapenem-non-susceptible *E. coli* isolates in 2010 and 2012, respectively. The genes encoding cabapenemases and plasmidic AmpC-type and extended-spectrum β-lactamases (EBSLs) were analyzed by polymerase chain reaction (PCR). The major porin channels OmpF and OmpC were evaluated by sodium dodecyl sulfate-polyacrylamide gel electrophoresis (SDS-PAGE). Molecular typing was performed with pulsed-field gel electrophoresis (PFGE) and multi-locus sequence typing (MLST).

**Results:**

The resistance rates of CnSEC isolates to cefazolin, cefotaxime, cefoxitin, ceftazidime, and ertapenem were all 100%, and most (94.7%) isolates were CMY producers. The main mechanism of CnSEC in Taiwan is via plasmidic AmpC β-lactamase CMY-2 and DHA-1 in combination with the loss of OmpC/F. In 2010, one isolate was confirmed to harbor *bla*_IMP-8_; a KPC-2 producer and an NDM-1 producer were detected in 2012. No isolate had VIM- or OXA-carbapenemases. ST131 was the predominant ST type (33.3%). PFGE revealed no large cluster in CnSEC isolates in Taiwan.

**Conclusions:**

The co-existence of plasmidic AmpC β-lactamase and outer membrane protein loss is the main mechanism for CnSEC in Taiwan. The emergence of KPC-2 and NDM-1 in 2012 and the predominance of ST131 warrant close monitoring and infection control.

## Background

*Escherichia coli* is an important member of Enterobacteriaceae that is evolving drug resistance mechanisms. Extended-spectrum β-lactamases (ESBLs) conferring resistance to extended-spectrum cephalosporins lead to treatment failure by penicillins and cephalosporins and are prevalent in *E. coli* isolates from hospital patients, community members, farm animals, and food. Carbapenems are powerful antibiotics that are not inactivated by ESBLs and AmpC β-lactamases and therefore are regarded as the last line of treatment for infections by ESBL producers. Carbapenem resistance in Enterobacteriaceae, particularly *Klebsiella pneumoniae*, has recently been reported worldwide, whereas carbapenem-resistant *E. coli* is relatively rare [1]. Nonetheless, carbapenem resistance in *E. coli* should be closely monitored because of its potential to spread in both hospital and community settings [2].

The mechanisms of carbapenem resistance in Enterobacteriaceae are the presence of either carbapenemase or a combination of β-lactamases and porin loss [3-7]. There are three types of carbapenemases that hydrolyze carbapenems, including class A (penicillinases), class B (metallo-β-lactamases), and class D (oxacillinase). Although IMP- and VIM-MBLs have typically been the most common types of MBLs found in Enterobacteriaceae [8], KPC (class A) enzymes are now the most predominant carbapenemases worldwide [9]. Carbapenemase-harboring *E. coli* is relatively less reported, though the first report of KPC-2 in *E. coli* was in 2006 [10]. This could be because carbapenem resistance does not occur naturally in *E. coli*[11], and it has been reported that plasmid transfer between *K. pneumoniae* and *E. coli* does not readily occur [12].

With regard to ESBL-producing *E. coli*, pandemic *E. coli* MLST type ST131 with CTX-M β-lactamase is spreading [13]; thus, whether the spread of carbapenem resistance in *E. coli* is related to certain clones warrants investigation. Accordingly, we initiated a nationwide surveillance program in 2010 and 2012 to investigate the molecular epidemiology of imipenem- or meropenem-non-susceptible *E. coli* in Taiwan.

## Methods

### Bacterial strains and susceptibility testing

Participating hospitals in the national survey identified imipenem- or meropenem-non-susceptible *E. coli* and sent all these isolates to a reference laboratory in National Health Research Institutes, Taiwan. A total of 32 imipenem- or meropenem-non-susceptible *E. coli* isolates were consecutively collected from eight hospitals participating in 2010 (five medical centers and three regional hospitals), with 43 collected from 17 hospitals (eight medical centers and nine regional hospitals) in 2012 [14]. All the isolates were from individual cases. This study was approved by the institutional review board of Kaohsiung Medical University Hospital (IRB No. KMUH-IRB-2 0130328). The isolates were obtained as part of routine hospital care procedures, and written informed consent for participation in the study was waived. The primary screening for carbapenem resistance was performed by the individual participating hospitals using the disc diffusion method. Species identification was performed by standard biochemical methods: the API system or the Vitek2 automated system. Further confirmation of antimicrobial susceptibility was determined by the broth micro-dilution method according to the guidelines of the Clinical and Laboratory Standards Institute (CLSI) [15]. In early 2012, CLSI revised the ertapenem breakpoints; thus, to compare the MIC results from different years, we used CLSI (M100-S22-U, 2011) [14] to interpret the ertapenem susceptibility results. For the susceptibility testing results, only isolates from the eight hospitals that participated in both 2010 and 2012 surveillance projects were analyzed and compared. The following anti-microbial agents were tested: cefazolin, cefotaxime, ceftazidime, cefepime, cefoxitin, ertapenem, imipenem, meropenem, doripenem, gentamicin, amikacin, ciprofloxacin, trimethoprim-sulfamethoxazole, colistin, and tigecycline. Quality control was performed using *E. coli* ATCC 35218 and ATCC 25922 reference strains.

### Detection of genes encoding carbapenemases, AmpC, and ESBLs

Carbapenemases (class B families, IMP, VIM, NDM, GIM, SPM, and SIM; class A families, NMC, IMI, SME, KPC, and GES; class D, OXA-48), plasmidic AmpC (CMY, DHA, and ACT) [16], and ESBL genes (CTX-M, TEM, and SHV) were detected by PCR amplification [17]. Primers for the class B carbapenemase NDM-1 were newly designed for this study (NDM-1F, TCTCGACATGCCGGGTTT; NDM-1R, GAGATTGCCGAGCGACTT). The amplicons were sequenced, and the entire sequence of each gene was compared to the sequences in the GenBank nucleotide database at http://www.ncbi.nlm.nih.gov/blast/.

### Transfer of *bla*_NDM-1_ and plasmid DNA analysis

Plasmid conjugation was performed using *E. coli* J53 AzR as the recipient strain. The recipients and *bla*_NDM-1_-carrrying donor samples were separately inoculated into brain-heart infusion broth and incubated at 37°C for 4 h. The samples were then mixed at a ratio of 10:1 (Donor:Recipient, by volume) for overnight incubation at 37°C. A 0.1-ml aliquot of the overnight broth mixture was spread onto a MacConkey agar plate containing sodium azide (100 μg/ml) and imipenem (1 μg/ml). The plasmids were extracted from these transconjugants using the standard alkaline lysis method, and fingerprints of the plasmids were generated by digestion with *Hinc*II or *Pvu*II (New England Biolabs, Beverly, MA).

### Pulsed-field gel electrophoresis (PFGE)

Total DNA was prepared, and PFGE was performed as described [18]. The restriction enzyme *Xba*I (New England Biolabs, Beverly, MA) was used at the temperature suggested by the manufacturer. The restriction fragments were separated by PFGE in a 1% agarose gel (Bio-Rad, Hercules, CA) in 0.5× TBE buffer (45 mM Tris, 45 mM boric acid, and 1.0 mM EDTA, pH 8.0) for 22 h at 200 V at a temperature of 14°C, with ramp times of 2–40 s using a CHEF Mapper apparatus (Bio-Rad Laboratories, Richmond, CA). The Dice coefficient was used to calculate similarities, and the unweighted pair-group method with the arithmetic mean method was used for the cluster analysis with BioNumerics software version 5.10 (Applied Maths, St-Martens-Latem, Belgium). Similarity PFGE patterns Hospital ST Cluster.

### Isolation and analysis of outer membrane proteins

**Figure 1:**
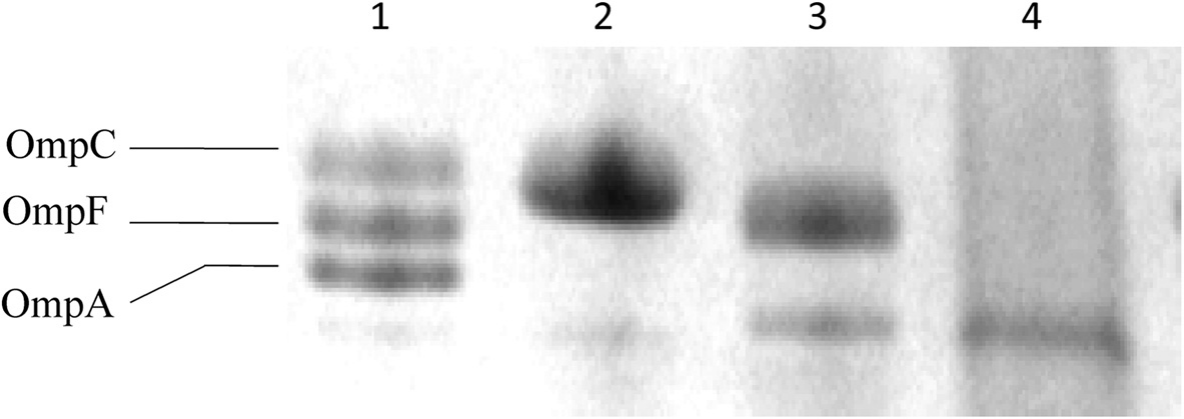
**Outer membrane profile of ATCC control isolates and clinical isolates of *E. coli *from this study.** Lane 1 shows the expression of OmpC, OmpF, and OmpA in the ATCC25922 control strain. Lane 2 shows OmpF deficiency; Lane 3 shows OmpC deficiency; Lane 4 shows both OmpF and OmpC deficiency.

Bacterial outer membrane proteins (OMPs) were prepared as described [19]. The OMPs were then separated by sodium dodecyl sulfate-polyacrylamide gel electrophoresis (SDS-PAGE) through 7.5% polyacrylamide-6 M urea gels and visualized by Coomassie Blue staining (Bio-Rad). A reference strain, *E. coli* ATCC25922, was included as a control. Figure 1 shows representative SDS-PAGE results to illustrate the interpretation of OmpC and OmpF loss.

### Multi-locus sequence typing (MLST)

MLST with seven housekeeping genes [20], *adk* (adenylate kinase), *fumC* (fumarate hydratase), *gyrB* (DNA gyrase), *icd* (isocitrate dehydrogenase), *mdh* (malate dehydrogenase), *purA* (adenylosuccinate synthetase), and *recA* (ATP/GTP motif), was performed on all isolates according to the protocol described on the *E. coli* MLST website (http://mlst.ucc.ie/mlst/dbs/Ecoli/documents/primersColi_html). The allele sequences and sequence types (STs) were verified at the http://mlst.ucc.ie/mlst/dbs/Ecoli/ website.

## Results and discussion

All isolates were resistant to cefazolin, cefotaxime, ceftazidime, cefoxitin, and ertapenem. With the exception of amikacin, the rates of resistance to the other tested antimicrobial agents increased in 2012. For the eight hospitals that participated in both the 2010 and 2012 surveillance projects, the resistance rates of imipenem, meropenem, doripenem, and cefepime increased significantly (*P* < 0.05) (Table 1). Indeed, the resistant rates to imipenem, meropenem, and doripenem were 56.3%, 31.3%, and 15.6%, respectively, in 2010 and increased to 72.1%, 58.1%, and 51.2%, respectively, in 2012. All isolates were susceptible to tigecycline and colistin in 2010, whereas the resistant rates to tigecycline and colistin were 7.0% and 2.3%, respectively, in 2012.

**Table 1:** **Antimicrobial susceptibility testing results for carbapenem-non-susceptible *E. coli *** **isolates in 2010 and 2012**

**Antibiotics**	**ertapenem-non-susceptible *E. coli *** **(8 hospitals) and imipenem- or meropenem-non-susceptible *E. coli *** **(17 hospitals)**							
	**2010 (n = 32)**				**2012 (n = 43)**			
	**MIC range (μg/ml)**	**MIC**_**50 **_**(μg/ml)**	**MIC**_**90 **_**(μg/ml)**	**Resistance (%)**	**MIC range (μg/ml)**	**MIC**_**50 **_**(μg/ml)**	**MIC**_**90 **_**(μg/ml)**	**Resistance (%)**
Ertapenem	2- ≥ 8	≥8	≥8	100	1- ≥ 8	≥8	≥8	100
Imipenem	1- ≥ 8	4	≥8	56.3	1- ≥ 8	≥8	≥8	72.1
Meropenem	0.12- ≥ 8	2	8	31.3	0.5- ≥ 8	4	≥8	58.1
Doripenem	0.12- ≥ 4	1	4	15.6	0.5- ≥ 4	≥4	≥4	51.2
Amikacin	≤4-64	4	32	6.3	≤4-32	≤4	16	0
Gentamicin	≤1- ≥ 16	2	≥16	40.6	≤1- ≥ 16	4	≥16	45.7
Cefazolin	≥32	≥32	≥32	100	≥32	≥32	≥32	100
Cefotaxime	4- ≥ 64	≥64	≥64	100	32- ≥ 64	≥64	≥64	100
Cefoxitin	≥32	≥32	≥32	100	≥32	≥32	≥32	100
Ceftazidime	16- ≥ 32	≥32	≥32	100	≥32	≥32	≥32	100
Cefepime	0.25- ≥ 32	8	≥32	18.8	2- ≥ 32	16	≥32	46.5
Ciprofloxacin	0.06- ≥ 4	≥4	≥4	75.0	≤0.06- ≥ 4	≥4	≥4	79.1
Tigecycline	≤0.25-1	≤0.25	0.5	0	≤0.25- ≥ 4	≤0.25	0.5	7.0
Colistin	≤0.5-2	1	2	0	≤0.5- ≥ 4	≤0.5	1	2.3
SXT^*a*^	0.06- ≥ 16	≥16	≥16	62.5	≤2/38- ≥ 4/76	≥4/76	≥4/76	76.7

^*a*^SXT: Trimethoprim/sulfamethoxazole. Trimethoprim/sulfamethoxazole MICs are presented according to the concentration of trimethoprim in 2010.

**Figure 2:**
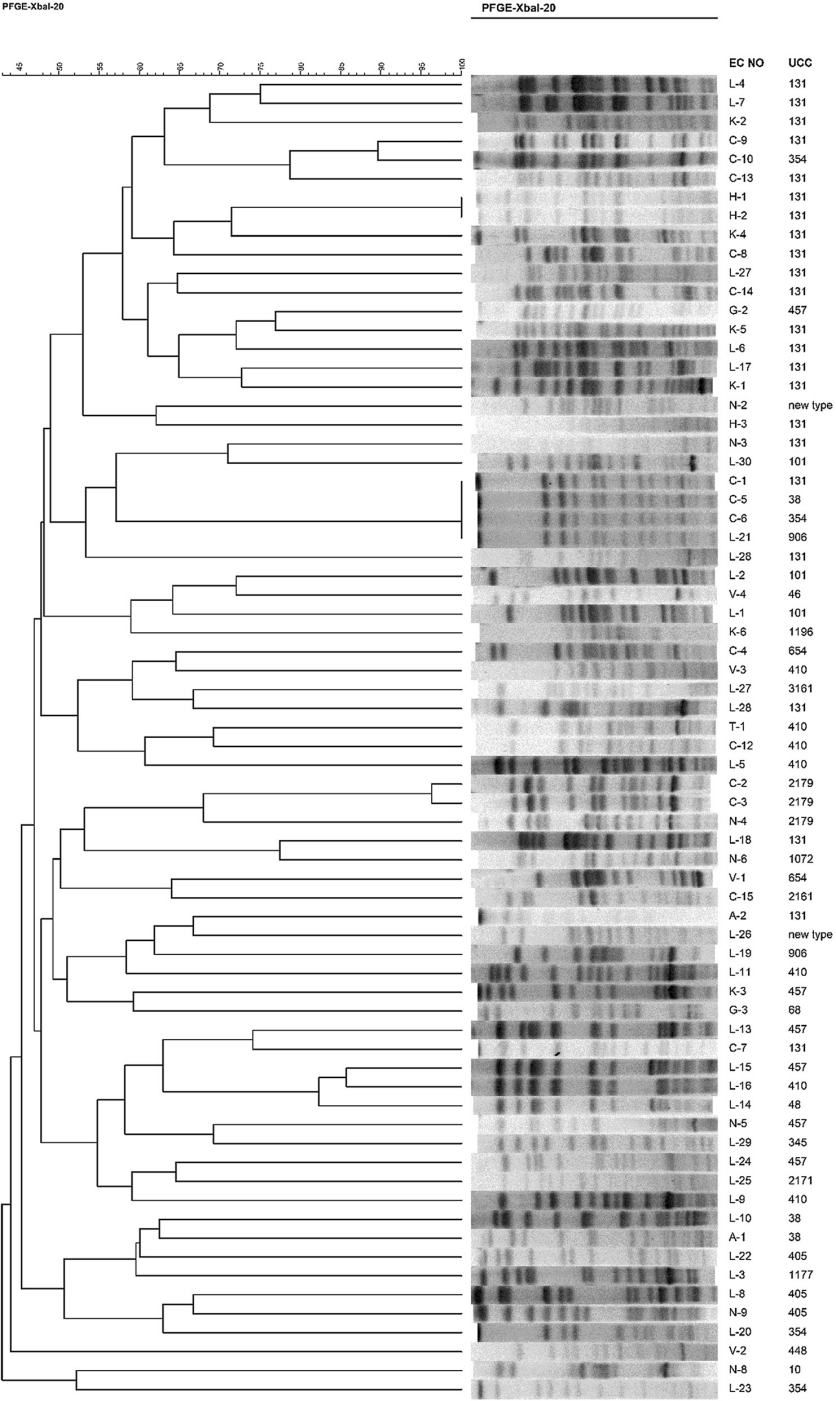
**A dendrogram of the pulse-field gel electrophoresis (PFGE) fingerprinting results and the ST types of 70 typable *E. coli *** **isolates.**

Of the 75 *E. coli* isolates, five were not typable by PFGE. No major cluster was found, except for two pulsotypes with 4 and 2 isolates each (Figure 2). The PFGE results suggested that clonal spread was not responsible for the emergence of carbapenem-resistant *E. coli*.

Based on MLST, ST131 was found to be the major sequence type. Of the 75 *E. coli* isolates, ST131 was detected in 25 isolates (9 isolates in 2010 and 16 isolates in 2012). Seven isolates were assigned to ST410, and six isolates were assigned to ST457. ST405 and ST101 were found in three isolates each. The remaining isolates exhibited diverse ST types. The isolates of MLST type ST131 showed no significant difference in distribution in 2010 and 2012 and displayed highly diverse PFGE patterns.

In 2010, all the isolates were negative for KPC-, NDM-, VIM-, and OXA- carbapenemases, though one isolate was confirmed to harbor *bla*_IMP-8_. In 2012, one KPC-2-producing isolate and one NDM-1-producing isolate were detected, whereas no IMP-, VIM-, and OXA-type carbapenemases were found.

In Asia, KPC-producing Enterobacteriaceae was first detected in *K. pneumoniae* in China in 2004 [21] and was also soon found in *Citrobacter freundii, E. coli,* and *Serratia marcescens*[22,23]. In Taiwan, the first three KPC-2-positive patients were detected in *K. pneumoniae* isolated from patients having been hospitalized in China [24,25]. Since then, KPC-2-producing *K. pneumoniae* has been increasingly reported in Taiwan [26], though few cases of KPC-positive *E. coli* have been reported [27]. In the present report, we identify the first KPC-2-positive *E. coli* from Taiwan. This KPC-2-containing *E. coli* was obtained from a 78-year-old female who had not been abroad but was transferred from a regional hospital to a middle Taiwan medical center for a urinary tract infection. The KPC-2-containing isolate also produced TEM-1, CMY-2, and CTX-M-3/CTX-M-22 β-lactamases and was classified as MLST sequence type 410 (ST410). To date, KPC-producing *E. coli* has only been reported in Israel, the USA, China, Brazil, France, Greece, and Ireland [10,12,22,28-31], and two major sequence types containing KPC-carbapenemases have been reported. ST131 was reported in France [31], the USA [27], and Ireland [30]; ST410 was reported in Greece [10] and Israel [1] (ST471 in the Pasteur Institute typing system is the same as ST410 in the UCC typing system). The possibility that ST131 and ST410 are future epidemic clones of KPC-2-producing *E. coli* worldwide is of great concern.

**Figure 3:**
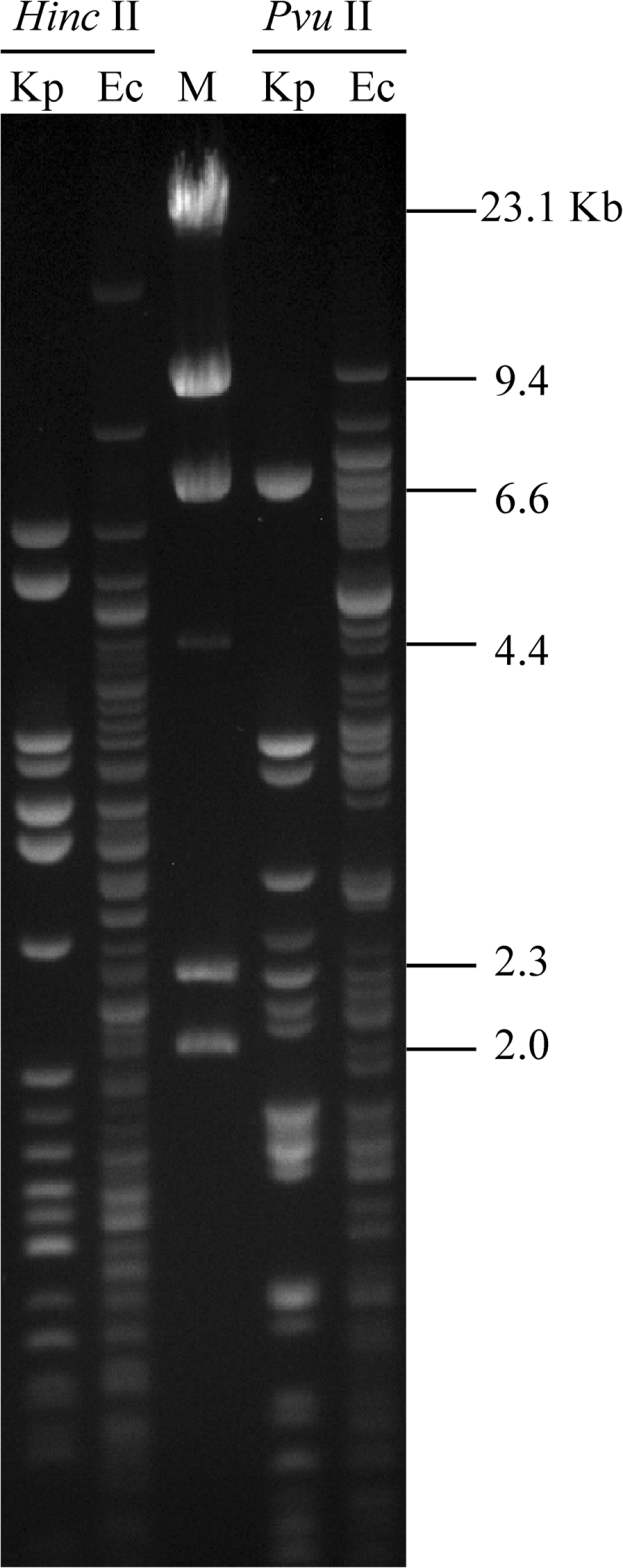
***Hinc*** **II- or *Pvu*** **II-digested plasmid profiles of transconjugants of NDM-1-carrying *K. pneumoniae *** **and *E. coli*.**

We also report the first NDM-1-harboring *E. coli* isolate in Taiwan. Interestingly, an NDM-1-positive *K. pneumoniae* was detected in the same hospital during the same period and was included in the study to compare the two NDM-1-containing plasmids. The two NDM-1 plasmids from *K. pneumoniae* and *E. coli* were successfully transferred to the recipient isolate *E. coli* J53. Additionally, the plasmid digestion profiles of the two NDM-1 plasmids revealed different patterns (Figure 3), indicating that plasmid spread is less likely. Our *E. coli* NDM-1 producer possessed TEM-1 and CMY-2 but did not encode CTX-M β-lactamases and 16S rRNA methylase genes, which are commonly found in *bla*_NDM-1_-positive isolates [32]. Thus far, ten sequence types (ST101, ST405, ST648, ST90, ST410, ST156, ST131, ST167, ST224, and ST38) of *E. coli* have been reported to contain NDM-1 [33]. However, the ST type of the NDM-1 clone described herein is ST345, which has not yet been reported to harbor NDM-1. This finding supports a conclusion that *bla*_NDM-1_ occurs in *E. coli* belonging to diverse phylogenetic lineages [34] and also emphasizes the need to study the plasmids carrying *bla*_NDM-1_ in *E. coli*.

All 75 isolates carried AmpC β-lactamase CMY and/or DHA-1; most of the isolates produced CMY-2 (68 isolates, 90.7%), and one CMY-4-carrying isolate was detected in 2012. DHA-1 was detected in six isolates in 2010 and one isolate in 2012. CTX-M-type ESBLs were detected in 13 isolates (40.6%) in 2010 (ten isolates exhibited CTX-M-14, and three each exhibited the CTX-M-14/CTX-M-15, CTX-M-14/CTX-M-55, or CTX-M-27/CTX-M-55 combinations), and ten isolates (23.3%) harbored CTX-M genes in 2012 (three isolates harboring CTX-M-14, three with CTX-M-79, two with CTX-M-15, and one each with the CTX-M-14/CTX-M-79, CTX-M-3/CTX-M-15 combination).

The 75 carbapenem-non-susceptible *E. coli* isolates showed the loss of at least one porin, whereas 42 (56%) isolates had lost both OmpC and OmpF. Sixteen (21.3%) isolates exhibited OmpF loss, and ten (13.3%) isolates exhibited OmpC loss.

Our study revealed that the main mechanism of imipenem or meropenem non-susceptibility in Taiwan is AmpC β-lactamase CMY-2 and DHA-1 in combination with OmpC/F loss (Table 2). It has been previously verified that such a combination of mechanisms can contribute to carbapenem resistance [6,7]. Most of the isolates were CMY producers (94.7%), and this most common CMY enzyme (CMY-2) of carbapenem-non-susceptible *E. coli* is also frequently found in cephamycin-resistant *E. coli* in community-acquired urinary tract infections in Taiwan [35].

**Table 2:** **Outer membrane profiles for carbapenem-non-susceptible *E. coli *** **isolates in 2010 and 2012**

**β-lactamases**	**Outer membrane profile**^***a***^							
	**2010 (8 hospitals; n = 32)**				**2012 (17 hospitals; n = 43)**			
	**F/C**	**△C**	**△F**	**△C/F**	**F/C**	**△C**	**△F**	**△C/F**
Carbapenemase								
KPC-2	0	0	0	0	1	0	0	0
NDM-1	0	0	0	0	0	0	1	0
IMP-8	0	0	0	1	0	0	0	0
AmpC only								
CMY	1	4	10	12	3	6	5	27
DHA	0	0	1	3	0	0	0	0
ESBL								
CTX-M	0	0	0	0	0	0	0	0
SHV	0	0	0	0	0	0	0	0

^*a*^Most isolates have multiple β-lactamase genes. The priority for the determination of β-lactamase in this table is carbapenemases, AmpC enzymes, and then ESBLs. No duplicate calculation was performed for each isolate.

*E. coli* sequence type ST131 is a pandemic clone associated with the CTX-M-15 gene and is resistant to fluoroquinolones [36]. Clone ST131 potentially harbors a variety of β-lactamase genes, most commonly CTX-M β-lactamases, followed by TEM, SHV, and CMY β-lactamases [13], and its dissemination has contributed to the spread of CTX-M-15-producing *E. coli*[37]. Of the 75 carbapenem-non-susceptible *E. coli* isolates, 25 (33.3%) were the ST131 type; of these, 23 isolates displayed a resistant to ciprofloxacin. However, most of the isolates (18/25, 72%) did not produce CTX-M-type β-lactamases. Thus, our results showed that *E. coli* ST131 is the predominant ST type of carbapenem non-susceptible *E. coli* in Taiwan but that it is not highly associated with CTX-M ESBLs. Our findings are similar to the results of Kim et al. [27] and support the conclusion that the ability of the ST131 clone to acquire resistance genes under selective pressure could be high.

We found that the low prevalence of CTX-M-type β-lactamases was most likely linked to the identification of a low amikacin resistant rate in this study. Amikacin resistance mediated by 16S rRNA methylase (*armA* and *rmtB*) in Gram-negative bacteria is an emerging resistance mechanism [38], and our previous study found that the 16S rRNA methylase gene (*armA* and/or *rmtB*) and the CTX-M gene were located on the same plasmid [39]. The low prevalence of CTX-M-type β-lactamases and the low amikacin resistant rate suggested the lack of an endemic plasmid to transfer both *bla*_CTX-M_ and 16S rRNA methylase genes for carbapenem-non-susceptible *E. coli*.

## Conclusions

OmpC and/or OmpF deficiency combined with AmpC (major CMY-2 and DHA-1) was found to be the major mechanism for the development of carbapenem non-susceptibility in Taiwan. The dominant clone of carbapenem-non-susceptible *E. coli* in Taiwan is ST131, which has also been found worldwide, and the high prevalence of this ST type illustrates the epidemic potential of this clone. The introduction of KPC-2 and NDM-1 into *E. coli* in Taiwan warrants further monitoring with regard to the epidemiology of carbapenem resistance, the resistance mechanisms, and the potential epidemic clones.

## Competing interests

The authors declare that they have no competing interests.

## Authors’ contributions

ML and PLL contributed to the data analysis and wrote the primary draft of the manuscript. LKS, JCL, TLW, CPF, JTW, PLL, and YCC participated in the study design and experiments. PLL and YCC reviewed and revised the paper. All authors have read and approved the final manuscript.

## Pre-publication history

The pre-publication history for this paper can be accessed here:

http://www.biomedcentral.com/1471-2334/13/599/prepub
